# The Molecule Cloud - compact visualization of large collections of molecules

**DOI:** 10.1186/1758-2946-4-12

**Published:** 2012-07-06

**Authors:** Peter Ertl, Bernhard Rohde

**Affiliations:** 1 Novartis Institutes for BioMedical Research, Novartis Campus, CH-4056, Basel, Switzerland

**Keywords:** Molecule cloud, Visualization, Scaffold analysis, Chemical databases, Open source

## Abstract

**Background:**

Analysis and visualization of large collections of molecules is one of the most frequent challenges cheminformatics experts in pharmaceutical industry are facing. Various sophisticated methods are available to perform this task, including clustering, dimensionality reduction or scaffold frequency analysis. In any case, however, viewing and analyzing large tables with molecular structures is necessary. We present a new visualization technique, providing basic information about the composition of molecular data sets at a single glance.

**Summary:**

A method is presented here allowing visual representation of the most common structural features of chemical databases in a form of a cloud diagram. The frequency of molecules containing particular substructure is indicated by the size of respective structural image. The method is useful to quickly perceive the most prominent structural features present in the data set. This approach was inspired by popular word cloud diagrams that are used to visualize textual information in a compact form. Therefore we call this approach “Molecule Cloud”. The method also supports visualization of additional information, for example biological activity of molecules containing this scaffold or the protein target class typical for particular scaffolds, by color coding. Detailed description of the algorithm is provided, allowing easy implementation of the method by any cheminformatics toolkit. The layout algorithm is available as open source Java code.

**Conclusions:**

Visualization of large molecular data sets using the Molecule Cloud approach allows scientists to get information about the composition of molecular databases and their most frequent structural features easily. The method may be used in the areas where analysis of large molecular collections is needed, for example processing of high throughput screening results, virtual screening or compound purchasing. Several example visualizations of large data sets, including PubChem, ChEMBL and ZINC databases using the Molecule Cloud diagrams are provided.

## Background

One of the most typical tasks that a cheminformatics expert in pharmaceutical or agrochemical industry performs practically daily is analyzing and visualizing large collections of molecules. Typical areas, where this is needed are the analysis of the company compound archive and its enhancement by purchasing additional molecules from commercial compound providers, analysis of high-throughput screening results, design of combinatorial libraries, chemogenomics analysis of bioactivity data and many others [1]. But also researchers in academia are facing similar challenges when they need to process and visualize large molecular databases that have become freely available in the last few years [2] or were even generated in silico [3]. A number of methods to analyze and visualize large collections of molecules exist [4]. Among the most commonly used ones are various clustering techniques, dimensionality reduction approaches [5] or methods based on substructure analysis, particularly the analysis of molecule scaffolds [6]. But even if such sophisticated methods are applied, at the end it is necessary to visually inspect large tables of molecular structure diagrams.

**Figure 1:**
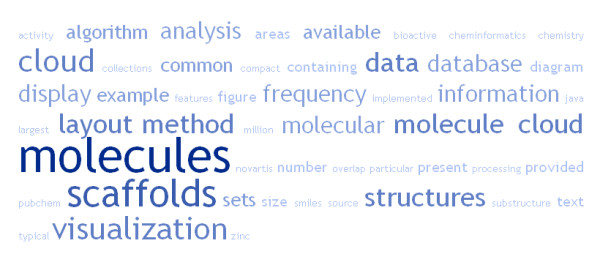
**Word cloud diagram created from the text of this article by interactive service **[16]**.**

The necessity to analyze and visualize large data sets, is of course, nothing specific to chemistry. Practically all areas of science need to cope with this problem. A simple, but very efficient method to analyze large data sets has been introduced in text processing. A method called “text cloud” or “word cloud” identifies the most common words in a text and displays them in form of a cloud diagram, where the frequency of a word is indicated by the size of the font used to render it. The most common words in the text like “and”, “or” or “the” do not contain useful information and are therefore not displayed. This type of diagram allows very efficient and compact visualization of the most important “message” contained in the text (Figure 1). The word cloud graphs summarizing the content of web pages become one of the symbols of Web 2.0. Several types of word cloud diagrams exist, differing in their layout and the way how the text is rendered.

The word cloud diagrams inspired us to develop a method for compact visualization of large molecular data sets. The method is based on the same principle, but instead of words molecular structures are displayed in the graph. The size of molecules corresponds to their frequency in the particular data sets. The largest structures catch the eyes of the observer first and therefore a single look at the diagram provides basic, condensed information about the content of the parent data set. In analogy with the “word cloud” we call this method ”Molecule Cloud”.

## Results and discussion

### Molecule Cloud methodology

The principle of the Molecule Cloud method is very simple. The most common substructure features present in the analyzed data set are identified and then displayed in such a way that their size corresponds to their frequency. The most common substructures are rendered the largest and therefore immediately catch the eye of an observer. Molecules are represented by their scaffolds, i.e. cores remaining when all non-ring substituents are removed. The concept of scaffold as the central part of a molecule is one of the basic concepts of medicinal chemistry and scaffolds play an important role in several drug discovery techniques like combinatorial chemistry and scaffold hopping [7]. Molecules without any rings are represented by their major chain, i.e. the longest chain, containing the largest number of heteroatoms. Reduction of molecules to scaffolds simplifies the analysis considerably. For example 35 million molecules from the PubChem database are represented by about 3.9 million scaffolds (50% of which are singletons, present only once in the database) and about 150 thousand chains.

Once the most common scaffolds and chains are identified, they need to be visualized with their size scaled according to the frequency in the parent database. It is well known that the frequency of various substructure features like scaffolds, substituents or linkers in molecular databases follows the power law (so called “long tail” distribution) [8]. This means that only few scaffolds in a database are very common, while there are many rare scaffolds including large number of singletons. Before using scaffold frequencies as a scaling factor, they therefore need to be transformed into the logarithmic scale. Benzene is a special case, practically in all large data sets the benzene is clearly the most frequent scaffold. In many cases it is therefore advisable not to display it. Even after logarithmic transformation of frequencies benzene would be disproportionally large, and it would not contribute any useful information. Removal of benzene is similar to the removal of the common stop words in classical text clouds.

According to our experience the optimal number of substructures to be displayed in the Molecule Cloud is between 100 and 250. This number usually contains 30 - 50 large structures, easily recognizable, the rest are smaller structures that optically fill the image. Of course, when displaying the graph in a larger area (for example as a poster) the number of structures that can be displayed is proportionally larger.

**Figure 2:**
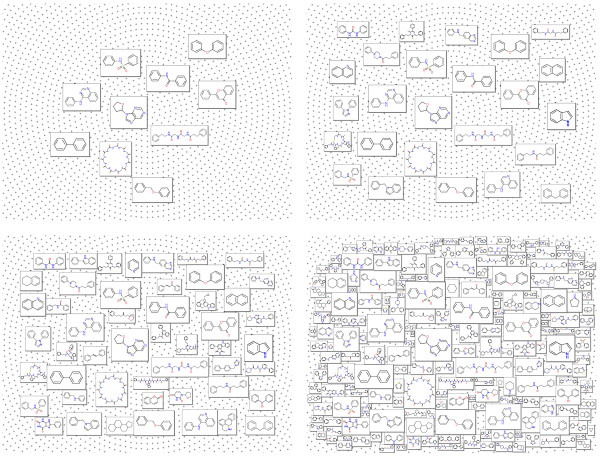
Illustration of the layout procedure.

The greatest challenge in generating the Molecule Cloud is the esthetically pleasing layout of molecules in the graph. This is done by a two-pass layout algorithm developed specially for this purpose. In the first step molecules are placed in the display area in such a way that their overlap is minimal. The process starts by sorting molecule images according to their size and placing the largest one in the center. Then other molecules, one by one are placed in a loop on a dense grid of predefined layout points and an “overlap score” is calculated for each placement. At the end the molecule is placed at the position with the best score (i.e. position where the overlap with other structures is minimal) and the next molecule is processed. This procedure is illustrated in the Figure 2, where one can see the layout after placement of 10, 25, 50 and 200 molecules. The “overlap score” used to identify the best position to place a molecule is calculated as the sum of overlap areas between molecular frames and the sum of the distances between molecule centers.

In most cases already this initial layout provides quite good placement of molecules. To further improve it, a second layout step is performed, namely an iterative optimization loop. In this step molecules, one by one are slightly moved in the direction that improves the total “overlap score”. The convergence is fast and after few seconds the final layout is achieved. During the optimization slight repulsive forces are also placed in the corners of the drawing area to provide aesthetically more pleasing “oval” display instead of completely filling the available image rectangle.

The Molecule Cloud layout algorithm has been implemented in Java. The layout itself does not require any “chemical intelligence”, it operates simply on the rectangles representing molecules. The required molecular processing capabilities, particularly parsing of SMILES and molecule depiction are defined by a Java interface class and may be implemented by using any cheminformatics toolkit. The program requires as input only a list of SMILES codes of structures to display with their frequencies and desired size of the final image as input. We tested the algorithm using two cheminformatics engines, the depiction engine from Molinspiration [9] and the recently released Novartis open source Avalon Cheminformatics Toolkit [10]. To interested parties the Java source code of the Molecule Cloud layout algorithm is available from the corresponding author under the terms of the BSD license. The distribution provides also instructions how to interface the program with the Avalon Cheminformatics Toolkit.

### Application examples

**Figure 3:**
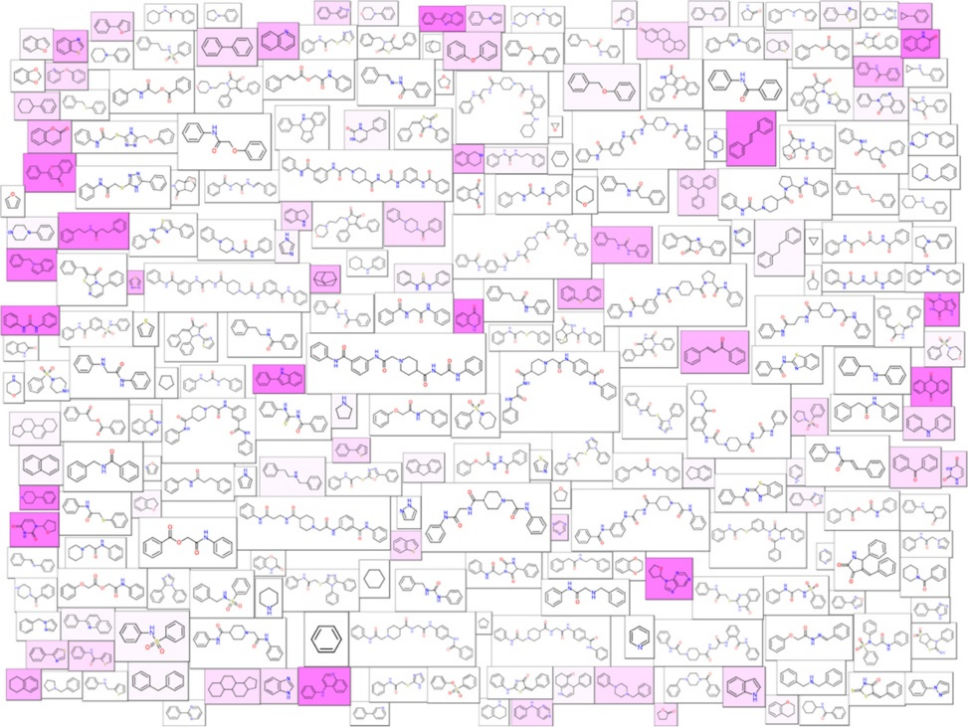
**Molecule Cloud for molecules in the PubChem database.** Magenta background indicates presence of biological activity.

In this section Molecule Cloud diagrams are presented for several popular publicly available data sets. PubChem [11] is the largest publicly available molecular structure database. In June 2012 it contained nearly 33 million unique structures. The Molecule Cloud of PubChem is shown in Figure 3. In this image, scaffolds of bioactive molecules are indicated by magenta background, where the color intensity is proportional to the ratio between bioactive and all molecules containing this scaffold. Bioactive molecules were identified by the PubChem advanced search as molecules having activity better than 10 μm in any PubChem assay.

**Figure 4:**
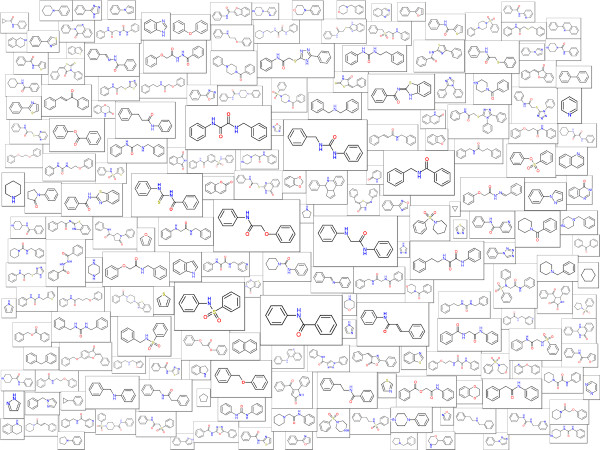
Molecule Cloud of the ZINC database.

A very useful resource for drug discovery, particularly for researchers in academia is the ZINC database [12]. ZINC, created and maintained by John Irwin from the University of California, San Francisco is a collection of commercially available compounds that may be used in virtual screening. The Molecule Cloud graph with the most common scaffolds present in about 12 million ZINC structures is shown in Figure 4.

**Figure 5:**
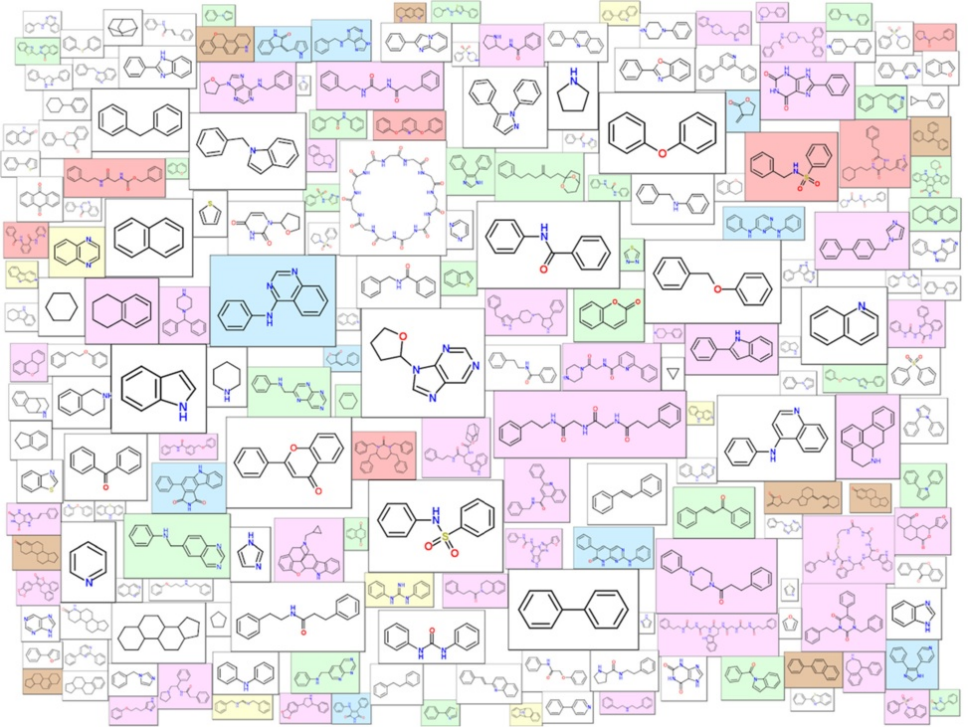
**Molecule Cloud of the ChEMBL database.** Preference of scaffold for particular target class is indicated by color (magenta - GPCRs, blue – kinases, red – proteases, green – other enzymes, brown – nuclear receptors, yellow – ion channels), non-colored scaffolds show activity on multiple targets.

The last example shows results of analyses of molecules in the ChEMBL database [13]. ChEMBL is a database of molecules extracted from medicinal chemistry journals and other sources. It also contains biological activities and information about the respective targets. This database is an extremely useful source of information particularly for scientists in academia, providing the type of information that was before available only to researchers in pharmaceutical industry. Molecule Cloud visualizing the most common scaffolds of more than 350,000 bioactive molecules (having activity below 10 μm) from ChEMBL is shown in Figure 5. In this figure, information about targets is also displayed by using color. The following six target classes were considered: GPCRs, ion channels, nuclear receptors, kinases, proteases and other enzymes. The scaffold box is colored if at least 70% of molecules containing this scaffold show activity on particular target class. Scaffolds exhibiting multiple types of activities are not colored. Among these, one can see several known “privileged scaffolds” [14] including biphenyl, indole, quinoline and purine.

**Figure 6:**
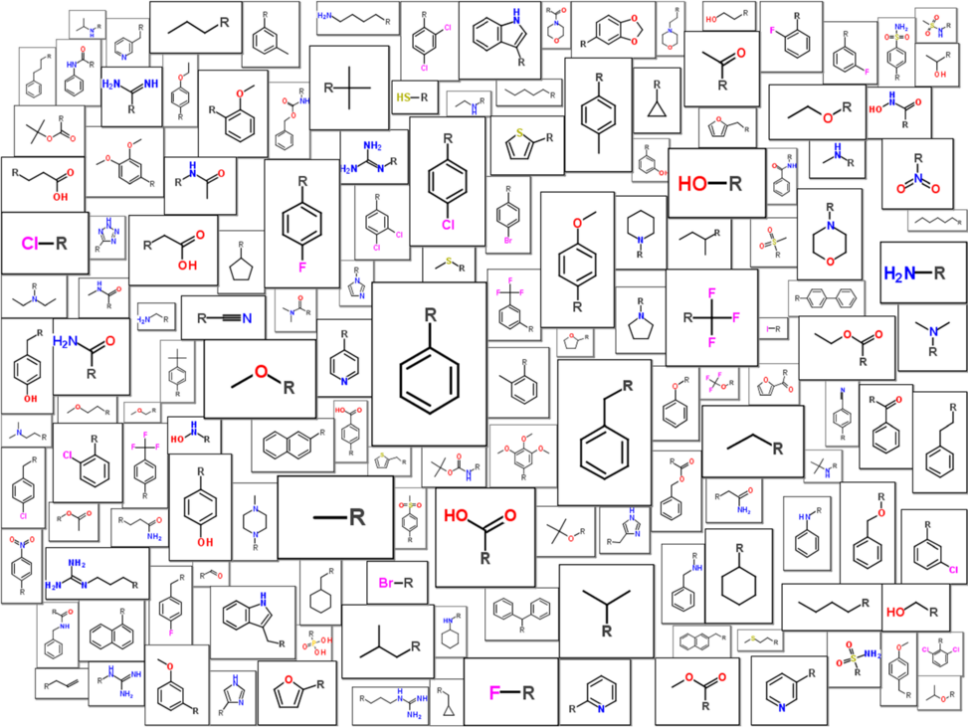
The most common substituents in the ChEMBL database.

The Molecule Cloud allows, of course, visualization of also other structural elements than scaffolds. This is illustrated in Figure 6, where the most common substituents (up to 15 atoms) from the ChEMBL database are shown. Although majority of the substituents are the same as in the other databases [15], the Molecule Cloud visualization provides clear advantage in comparison with classical molecule grid display.

## Conclusions

Molecule Cloud - a method for compact visualization of the typical substructures present in large collections of molecules was described here. The Molecule Cloud graphs allow to recognize scaffolds and other substructure features that are typical for particular data set by a single look. Additional information about molecules may be included by using colors. Further enhancement of generated images, for example by adding textual information, or possibility to click on particular scaffold to see the molecules containing it, should be with help of HTML5 technology easy.

**Figure 7:**
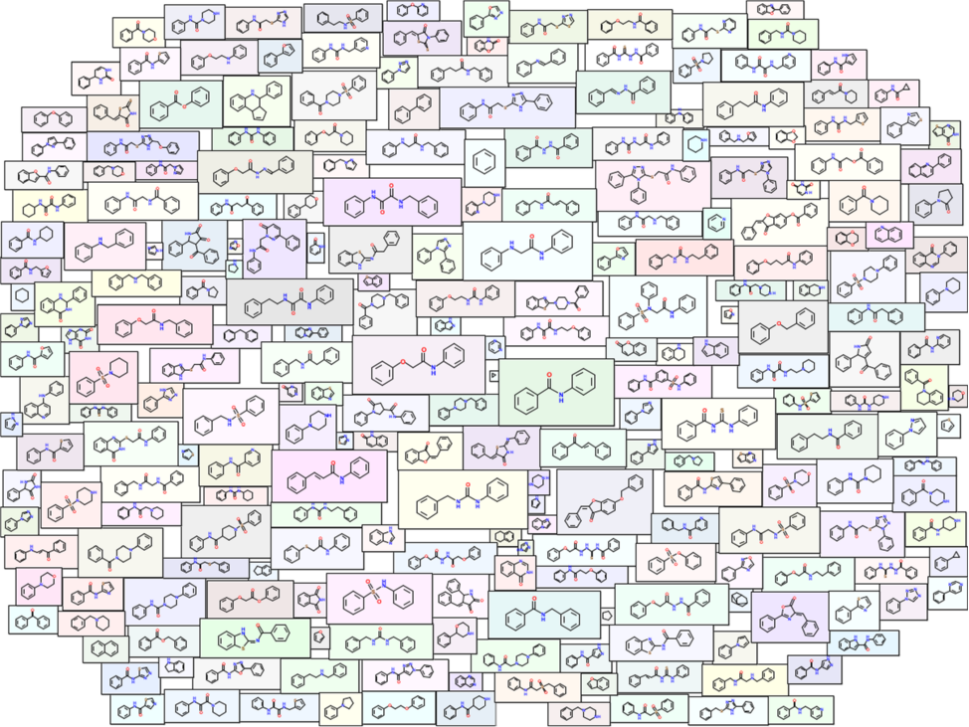
Molecule Cloud diagram of the ZINC database rendered by the Avalon Cheminformatics Toolkit.

The Java source code of the Molecule Cloud layout algorithm is available from the corresponding author on request. When combined with the recently released Avalon Cheminformatics Toolkit the software allows generation of Molecule Cloud diagrams (Figure 7) with a completely open source solution.

## Competing interests

The authors declare that they have no competing interests.

## Authors’ contributions

PE developed the idea to display set of molecules as a Molecule Cloud in analogy to the visualization of text information as a word cloud and implemented the layout algorithm in Java. BR is author of the Avalon Cheminformatics Toolkit and he interfaced this toolkit with the Molecule Cloud. Both authors read and approved the final manuscript.
